# CD4 T Cell Dependent Colitis Exacerbation Following Re-Exposure of *Mycobacterium avium* ssp. *paratuberculosis*

**DOI:** 10.3389/fcimb.2017.00075

**Published:** 2017-03-16

**Authors:** Abdulhadi Suwandi, Imke Bargen, Marina C. Pils, Martina Krey, Susanne Zur Lage, Anurag K. Singh, Tina Basler, Christine S. Falk, Ursula Seidler, Mathias W. Hornef, Ralph Goethe, Siegfried Weiss

**Affiliations:** ^1^Molecular Immunology, Helmholtz Centre for Infection ResearchBraunschweig, Germany; ^2^German Centre for Infection Research, Institute of Medical Microbiology and Hospital Epidemiology, Hannover Medical SchoolHannover, Germany; ^3^Mouse Pathology, Helmholtz Centre for Infection ResearchBraunschweig, Germany; ^4^Department of Gastroenterology, Hepatology, Endocrinology, Hannover Medical SchoolHannover, Germany; ^5^Institute for Microbiology, University of Veterinary Medicine HannoverHannover, Germany; ^6^Integrated Research and Treatment Center Transplantation, Institute of Transplant Immunology, Hannover Medical SchoolHannover, Germany; ^7^Institute of Medical Microbiology, RWTH University Hospital AachenAachen, Germany; ^8^Institute of Immunology, Hannover Medical SchoolHannover, Germany

**Keywords:** MAP, DSS, colitis, repeated infection, CD4+T cells

## Abstract

*Mycobacterium avium* ssp. *paratuberculosis* (MAP) is the causative agent of Johne's disease (JD), a chronic inflammatory bowel disease of cattle characterized by intermittent to chronic diarrhea. In addition, MAP has been isolated from Crohn's disease (CD) patients. The impact of MAP on severity of clinical symptoms in JD as well as its role in CD are yet unknown. We have previously shown that MAP is able to colonize inflamed enteric tissue and to exacerbate the inflammatory tissue response (Suwandi et al., 2014). In the present study, we analyzed how repeated MAP administration influences the course of dextran sulfate sodium (DSS)-induced colitis. In comparison to mice exposed to DSS or MAP only, repeated exposure of DSS-treated mice to MAP (DSS/MAP) revealed a significantly enhanced clinical score, reduction of colon length as well as severe CD4^+^ T cell infiltration into the colonic *lamina propria*. Functional analysis identified a critical role of CD4^+^ T cells in the MAP-induced disease exacerbation. Additionally, altered immune responses were observed when closely related mycobacteria species such as *M. avium* ssp. *avium* and *M. avium* ssp. *hominissuis* were administered. These data reveal the specific ability of MAP to aggravate intestinal inflammation and clinical symptoms. Overall, this phenotype is compatible with similar disease promoting capabilites of MAP in JD and CD.

## Introduction

*Mycobacterium avium* ssp. *paratuberculosis* (MAP) is the cause of Johne's disease (JD), a chronic inflammatory disorder of the gastrointestinal tract in ruminants. Clinical symptoms of the disease include chronic diarrhea and loss of body weight ultimately associated with a fatal wasting disease. Large numbers of bacteria are shed intermittently during active disease and the contamination of milk, soil, or water represents the mode of MAP transmission (Cocito et al., 1994). MAP exhibits high tenacity illustrated by its ability to survive the pasteurization process and may thus, to some extent, be present in the human food chain (Sung and Collins, 1998). The pathogenesis of JD is the still unclear. However, bacterial survival and proliferation in sub-epithelial macrophages appear to represent critical steps promoting the disease (Whittington et al., 2012).

An association of MAP infection with Crohn's disease (CD) a chronic inflammatory bowel disease in humans has been proposed based on the similarity of symptoms as well as histopathological appearance between JD in ruminants and CD in humans (Chiodini et al., 1984; Feller et al., 2007; Abubakar et al., 2008). Studies, comparing the presence of MAP in the intestine of CD patients, however, have provided contradictory results (Chiodini et al., 1984; Feller et al., 2007; Abubakar et al., 2008). This might be due to technical difficulties in the detection of MAP in clinical samples. For instance, cultivation of MAP on solid media may require months to years (Bull et al., 2003). On the other hand, the detection of MAP DNA by PCR, as observed in some CD patients, does not prove the presence of viable bacteria. Hence, the question whether MAP plays a causative or disease promoting role in CD or may only be a silent bystander still remains unanswered.

Macrophages and/or dendritic cells (DC) initiate the activation of the adaptive immune system and act as antigen presenting cells (APC). Upon MAP infection, they secrete cytokines such as IFN-γ, TNF-α, IL-6, and IL-1β that stimulate T lymphocyte activation. In turn, CD4^+^ T cells of T helper 1 type (Th1) are activated and produce IFN-γ. Such MAP specific CD4^+^ T cells home to the site of infection and are thought to restrict bacterial growth (Coussens, 2004). This is consistent with our previous data showing that systemically applied MAP elicits signs of systemic and mucosal inflammation and exacerbates an already existing intestinal inflammation (Koc et al., 2014; Suwandi et al., 2014). In addition, our previous data suggested that MAP infection preferentially colonizes inflamed intestinal tissue (Suwandi et al., 2014), which would explain the elevated MAP detection rate in clinical samples of CD patients reported in some studies (Feller et al., 2007). Still, additional so far undefined cofactors might promote inflammation and explain the outcome of studies that did not report a significant association of MAP with CD.

An immune-mediated disease promoting effect of MAP might also contribute to JD. Animals are usually infected by MAP early in life and exhibit a long disease course from the pre-clinical phase with intermediate diarrhea to the clinical phase with chronic diarrhea and wasting. It is not clear why chronically infected animals convert to the overt, clinical phase of JD. One possible explanation for disease aggravation might be seen as a consequence of the ongoing, recurrent exposure of immune cells to MAP or MAP components released from macrophages. Based on this hypothesis, we performed the present study. It analyzes the clinical outcome as well as immunological parameters during dextran sulfate sodium (DSS)-induced colitis in the absence or presence of repeated exposure to MAP. Interestingly, DSS treated mice exposed to MAP but not mice treated solely with DSS or MAP exhibited diarrhea as well as a significant reduction of colon length. Both, diarrhea and deep intestinal tissue inflammation are also found in JD and CD. CD4^+^ T lymphocytes were identified to drive disease progression *in vivo*. Additionally, MAP infection resulted in stronger inflammation in comparison to closely related *M. avium* ssp. *avium* and *M. avium* ssp. *hominisuis* infection. Our results clearly demonstrate that recurrent exposure to MAP contributes to the aggravation of inflammation and clinical signs via the stimulation of CD4^+^ T cells.

## Materials and methods

### Bacteria

*M*. *avium* ssp. *paratuberculosis* (MAP) strain DSM 44135 was grown on Watson Reid medium supplemented with mycobactin J (1 mg/L) at 37°C (Kuehnel et al., 2001). *M. avium* ssp. *avium* (MAA) strain DSM 44156 and *M. avium* ssp. *hominissuis* (MAH) strain 04A/1287 were grown in the same medium without mycobactin J.

### Mice

C57BL/6J mice were purchased from Janvier (Le Genest-saint-Isle, France). RAG2^−/−^ mice were bred at the animal facility of the Helmholtz Centre for Infection Research (HZI) under specific pathogen-free conditions (SPF). Female mice at the age of 8–10 weeks at the beginning of all experiments were used. Animal studies were carried out under good animal practice conditions in strict accordance with the German law for animal protection (Tierschutzgesetz, §7–9) performed under the approval of the ethics committee of the local authority: LAVES (Nr.:33.14.42502-04/090/08).

### Induction of DSS induced colitis and MAP infection

Four Pecentage DSS (35–50,000 kDa; MP Biomedical) was administered via the drinking water from day 1 until day 5. For infection, mice were intraperitoneally (i.p.) injected with 10^8^ MAP, MAA, or MHS in endotoxin free Dulbecco's phosphate-buffered saline (PAA) 2 days after DSS administration (*n* = 3–5 mice per group). Body weight was monitored 2–3 times weekly. For secondary challenge, mice were injected 5 weeks post infection (5wk p.i.). Mice were sacrificed on day 1 post-secondary challenge. The total weight of liver and spleen were measured and the organs were harvested for histology. The colon length was measured, a 1–2 cm section was then obtained for histology, and the rest was homogenized for plating after washing with PBS. To deplete CD4 and CD8 T cells, 150 μg/mL anti-CD4 antibody (Clone GK1.5), anti-CD8 (Clone 53–6.7), or Rat IgG2b isotype control (eBR2a, eBioscience) as control were administered i.p. 2 days before secondary challenge.

### Measurement of fluid absorption in colon

Fluid absorption measurement was performed at Department of Gastroenterology, Hepatology, and Endocrinology, Hannover Medical School according to previously published protocols and described (Singh et al., 2010, 2012). Animals (*n* = 5 each group) had free access to food and water before the experiment. Induction of anesthesia was achieved by spontaneous inhalation of isoflurane (Forene; Abbott Germany, Wiesbaden, Germany). The inhalation gas contained a mixture of ~10–15% oxygen, ~85–90% air, and ~2.0 ± 0.2% isoflurane with the use of an isoflurane pump (Univentor 1250 Anaesthesia Unit; AgnTho, Lidingö, Sweden) and was administered continuously via a breathing mask. The depth of the anesthesia was tested by probing the pedal withdrawal reflex and breathing rate of the mice. The gas flow through the cylindrical mask (2 cm long with an inner diameter of 1.2 cm) was ~200 ml/min, which minimized rebreathing of exhaled CO_2_ by the animals. To minimize the anesthesia level and pain during surgery, all the mice received subcutaneous injection of 500 mg/kg body weight of Novalgin 30 min before the surgery was started. Body temperature was maintained using a heating pad. A catheter was placed in the left carotid artery for continuous infusion of (in mM) 200 Na^+^ and 100 ${\text{CO}}_{3}^{-2}$ at a rate of 0.30 ml/h to prevent anesthesia induced acidosis. Lower abdomen was opened by one small central incision, and whole colon was used for measurement. A small polyethylene tube (PE100) with a distal flange was advanced into the proximal colon through an opening in cecum and secured by a ligature that served as an inlet tube. PE200 flanged tubing was secured by ligature to allow for drainage through the anus in the distal part of the colon. The isolated colonic segment with an intact blood supply was gently flushed and then perfused (Perfusor compact; BRAUN, Bethlehem, PA, USA) at a rate of 3 ml/h with 150 mmol/l NaCl. Effluents from the isolated segment were visually free of blood throughout all experiments. After an initial 30-min washout and recovery period, fluid absorption was measured for another 1 h. At the end of the experiments, mice were killed by cervical dislocation. The perfused segment was excised from *in situ* position, and its length was measured. The perfusate was collected in a pre-weighed 4.5-ml collecting tube. After a 30-min period, the tube was weighed again and the difference of the two up to four places of decimal was taken as the amount of fluid recovered after 30 min (taking density of fluid roughly at about 1 g/ml). The difference was further subtracted from 1.5 ml, which was the original volume recovered after 30 min in case of no fluid absorption. All values were represented in milliliters of fluid absorbed per centimeter colon length per h (ml/cm/h).

### Pathology and histology

Histology was performed in the Mouse Pathology platform at HZI Braunschweig. Organs were fixed in 10% formaldehyde, dehydrated with ethanol, and embedded in paraffin. Paraffin sections (3 μm) were stained with hematoxylin-eosin (H&E) and Ziehl Neelsen (ZN) staining according to standard laboratory procedures. Scoring of histopathological changes of colon and ZN staining evaluation was performed in a blinded fashion (*n* = 3–5 mice per group). For scoring of colonic tissue, a combined score of severity, ulceration, edema, and area involved was applied. Grades applied for severity were 0 = no alteration, 1 = mild, 2 = moderate, 3 = severe alterations. For ulceration score, the grading was 0 = no ulcer, 1 = 1–2 ulcers (involving up to a total of 20 crypts), 2 = 1–4 ulcers (involving a total of 20–40 crypts) and 3 = any ulcers larger than 40 crypts. For edema score, the grades were 1 = only mild epithelial or submucosal edema, 2 = mild epithelial edema associated with mild submucosal edema or more moderate submucosal edema, and 3 = every edema more extensive that the previous. For area involved in the inflammatory process, the grades were 0 = 0%, 1 ≤ 30%, 2 = 40–70%, 3 ≥ 70%.

### ELISA

Colons were flushed with cold PBS, opened along longitudinal axis, cut into 0.5 cm pieces and incubated for 24 h in RPMI 1640 supplemented with 10% FCS and antibiotics (*n* = 3–5 mice per group). Colon supernatants were collected and stored at −20°C. TNF-α serum levels were detected using a mouse TNF-α ELISA kit (Biolegend) according to the manufacturer's protocol. Mouse cytokines and chemokines were analyzed using Bio-plex Pro Mouse Cytokines 23-plex Assay (Bio-Rad) according to the manufacturer's protocol. IFN-γ levels in sera or colon supernatant were measured using an ELISA. Rat anti-mouse IFN-γ (Molecular Immunology, HZI) in coating buffer were incubated in 96 well plates (MaxiSorb TM Immunoplates, Nunc) overnight at 4°C. The 96 well plates were then blocked for 1 h, with 3% BSA in 0.05% Tween 20. Diluted sera or colon supernatant were distributed to the wells and incubated for 2 h at room temperature. IFN-γ was detected with biotinylated anti-mouse IFN-γ antibodies (Molecular Immunology, HZI). Biotinylated antibodies were bound with horseradish peroxidase (HRP) conjugated streptavidin (BD Pharmingen). Bound HRP was determined using o-Phenylendiamin (OPD) as substrate and the results were read using an ELISA-reader (BioRad 3550-UV microplate reader) at a wavelength of 490 nm.

### Flow cytometry

Isolation of cells from mesenteric lymph nodes and colonic *lamina propria* was performed as described in Zigmond et al. (2012) with slight modifications (*n* = 3–5 mice per group). Cells from mesenteric lymph nodes were prepared by mechanical dissociation. Cell suspension was passed through 50 μm nylon filters. Red blood cells were lysed for 2 min in ACK buffer (0.15 M NH_4_Cl, 10 mM KHCO_3_, and 0.1 mM EDTA). FcR were then blocked with 1 μg/mL FcR block (rat anti-mouse CD16/CD32, BD Pharmingen).

Colons were flushed of their luminal content with cold PBS, opened longitudinally, cut into 0.5 cm pieces and incubated 3x in HBSS (Ca and Mg free) with 5% FCS, 2 mM EDTA, 1 mM DTT and 10 mM HEPES at 37°C with shaking at 250 rpm for 15 min to remove epithelial cells and mucus. Tissue was then digested in RPMI 1640 with 10% FCS, 1.5 mg/mL collagenase type VIII, and 0.1 mg/mL DnaseI at 37°C with shaking at 250 rpm for 45 min. Percoll (GE Healthcare) gradients using 40/80% were performed to purify the leukocytes. The following antibodies were used for flow cytometry analysis: anti-CD45 APC-Cy7, anti-CD3 FITC, anti-CD4 APC, anti-CD11c FITC, anti-CD19 PerCP-Cy5.5, anti-CD8 PE, anti-Ly6C APC, anti-Ly6G PE-Cy7, anti-F4/80 PerCP-Cy5.5, and anti-CD11b PE. DAPI (Sigma) were used for live and dead discrimination. Flow cytometry was performed using a LSR II analyzer (BD, NJ, USA). The data were analyzed using FACSDiva software (BD) or FlowJo (TreeStar).

### Organ plating

The mixture of colon and small intestine homogenates (*n* = 3–5 mice per group) were plated on Middlebrook 7H10 Agar (Difco, Heidelberg, Germany) containing Mycobactin J (IDVet Innovative Technology, Montpellier, supplemented with antibiotics [Vancomycin (Roth, Karlsruhe, Germany); Amphotericin B (Roth); and Nalidixin Acid (Sigma, Munich, Germany)]. The plates were incubated at 37°C for up to 8 weeks.

### Statistics

All data were analyzed with GraphPad Prism software. In some figures, statistical differences between groups were determined by One-way Anova followed by Tukey's multiple comparison post-test. The data were considered statistically significant when *p*-values were < 0.05. ^*^p < 0.05, ^**^p ≤ 0.01, ^***^p < 0.001.

## Results

### Repeated MAP exposure of DSS treated mice leads to diarrhea and aggravates colitis

**Figure 1:**
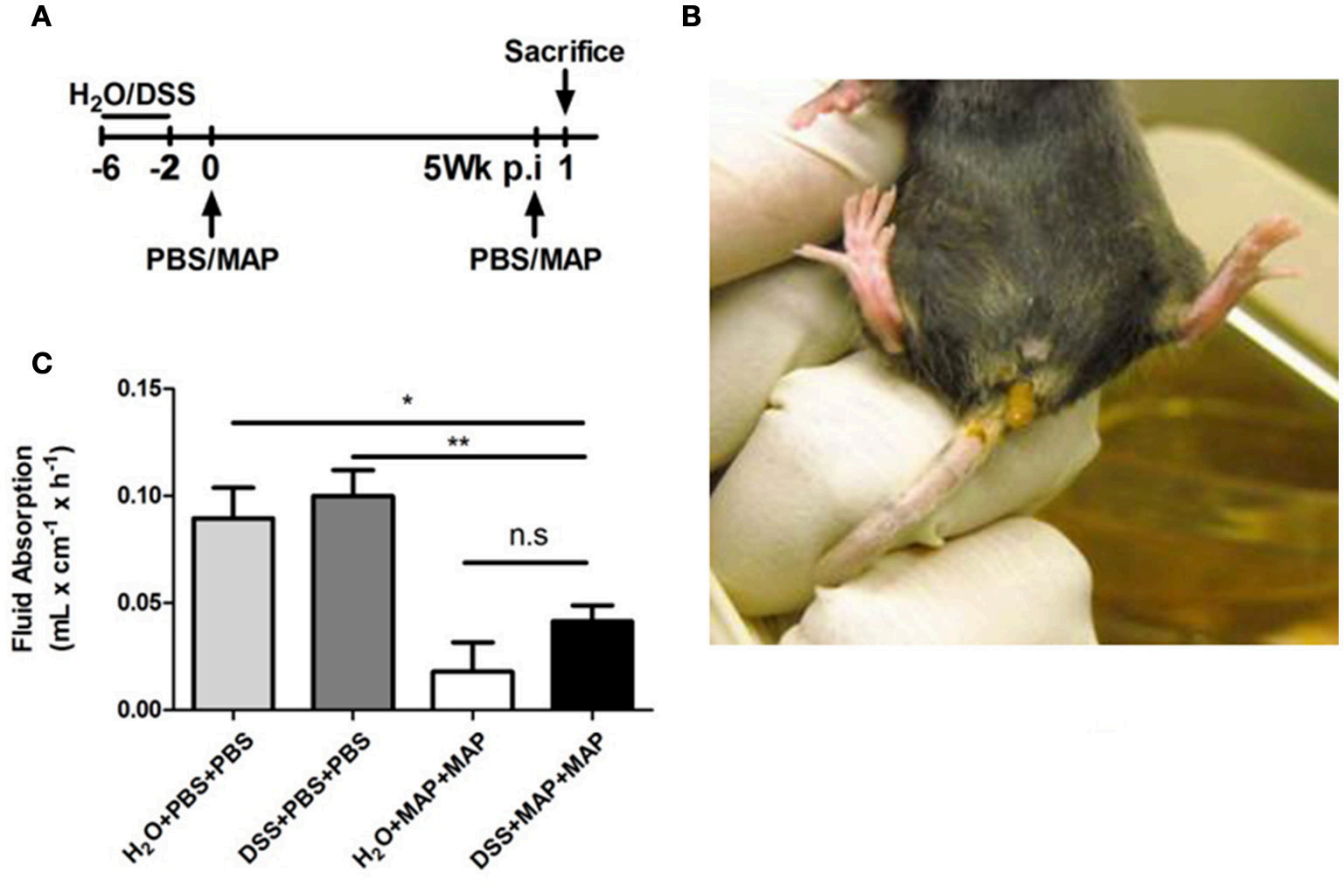
**MAP secondary infection leads to diarrhea and colitis. (A)** Overview of the experimental schedule. **(B)** Representative mouse with diarrhea at 1 day after secondary exposure. **(C)** Fluid absorption from colon in mL × cm^−1^ × h^−1^ (*n* = 5). Graph show a show as Mean ± SEM. ^*^P < 0.05; ^**^p < 0.01, one-way ANOVA with Tukey's multiple comparison post-test.

We have previously observed that i.p. infection of DSS-treated mice with MAP enhances the preexisting inflammatory response (Suwandi et al., 2014). In the present study, by using repeated MAP exposure we investigated the underlying mechanisms of the MAP-mediated exacerbating effect and its influence on the clinical outcome of an intestinal inflammation. Since persistent or recurrent exposure with MAP is thought to occur under natural conditions, DSS pretreated mice were exposed to MAP twice during the course of 5 weeks. Four percentage DSS was administered *via* the drinking water to adult female C57BL/6 mice from day −6 until day −2. Following 2 days of recovery, 10^8^ colony forming units (CFU) MAP were injected intraperitoneally. MAP exposure was repeated 5 weeks after the first administration as illustrated in the experimental schedule (Figure 1A). The 5 week interval was chosen to assure that mice had recovered from the first round of DSS treatment and MAP infection. In addition, we noticed that the severity of the reaction was weaker when the interval was shortened (data not shown).

First, the body weight was monitored (Figures S1A–D). DSS-treated mice exposed to MAP twice (DSS+MAP+MAP) showed a significantly enhanced body weight reduction at day 1 post secondary exposure and delayed subsequent weight gain in comparison to all other tested conditions. In contrast, DSS-treated mice infected with MAP only once (DSS+PBS+MAP) showed a reduction of body weight at 1 day p.i. but recovered already at day 2 p.i. (Figure S1D). Although the impact on body weight was not as severe, extended recovery times were also observed in mice infected twice with MAP without prior DSS treatment (H_2_O+MAP+MAP; Figure S1D).

Unexpectedly, mice that were pretreated with DSS and infected twice with MAP (DSS+MAP+MAP) exhibited transient diarrhea (Figure 1B). This phenomenon was observed in at least two of three experiments. Thus, in the presence of a preexisting mucosal damage, MAP elicits a more severe tissue reaction. In addition, repeated MAP exposure (H_2_O+MAP+MAP and DSS+MAP+MAP) resulted in a reduction of fluid absorption in the colon leading to clinically overt intestinal disease, irrespective of a preexisting tissue damage (Figure 1C).

**Figure 2:**
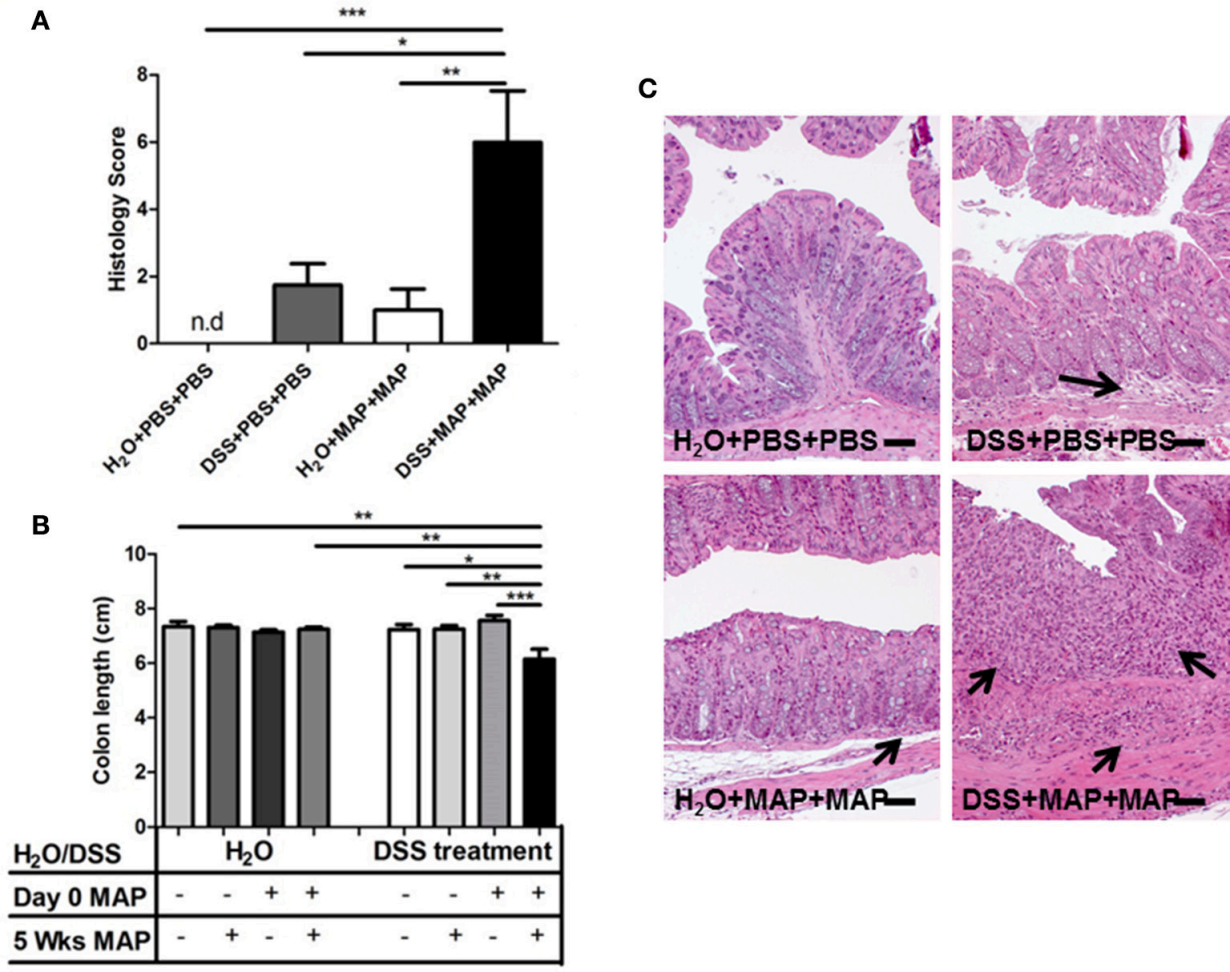
**Pathology of colon after exposure of DSS pretreated mice to MAP. (A)** Histology score of the colon was established at 1 day after secondary exposure (*n* = 3–5). n.d, not detected, histology score = 0. **(B)** Upon the necropsy at 1 day after secondary exposure, colon length was measured. Colon lengths in cm (*n* = 3–5). **(C)** H&E staining colon tissue section at 1 day after secondary exposure. Scale bars, 25 μm. H_2_O+PBS+PBS (upper left) showed normal tissue. H_2_O+MAP+MAP (bottom left) and DSS+PBS+PBS (upper right) showed mild infiltration of inflammatory cells (black arrow). DSS+MAP+MAP (bottom right) showed high invasion of inflammatory cells (black arrow) and severe epithelial hyperplasia. Graph show a representative of at least two independent experiments, show as Mean ± SEM. ^*^P < 0.05; ^**^p < 0.01; ^***^p < 0.001, one-way ANOVA with Tukey's multiple comparison post-test.

A reduction of the total colon length was detected only in DSS+MAP+MAP mice consistent with a significantly enhanced, synergistic tissue inflammation (Figure 2B). Also, the histological score of colons from DSS+MAP+MAP mice was significantly higher than the score found in control animals (Figure 2A). At that time point, H&E staining of colonic tissue obtained from DSS+MAP+MAP animals revealed strong infiltration of inflammatory cells, whereas for instance the tissue of DSS+PBS+PBS animals showed only a low degree of inflammatory cell infiltration (Figure 2C). Together, the development of diarrhea, the reduction of colon length and the enhanced histological score of the mucosal tissue in DSS+MAP+MAP mice indicate that repeated MAP administration significantly influences the course of DSS-induced mucosal tissue damage.

### Elevated cytokine levels after secondary MAP administration

A marked tissue reaction and weight loss was observed after secondary infection with MAP. In order to test whether the systemic influence was induced by soluble mediators, sera were collected at 6 h after secondary MAP exposure and the cytokine and chemokine levels were measured by multiplex analysis. Most cytokines and chemokines tested were strongly up-regulated in both groups following repeated MAP exposure, i.e., DSS+MAP+MAP and H_2_O+MAP+MAP mice (Supplementary Table 1).

**Figure 3:**
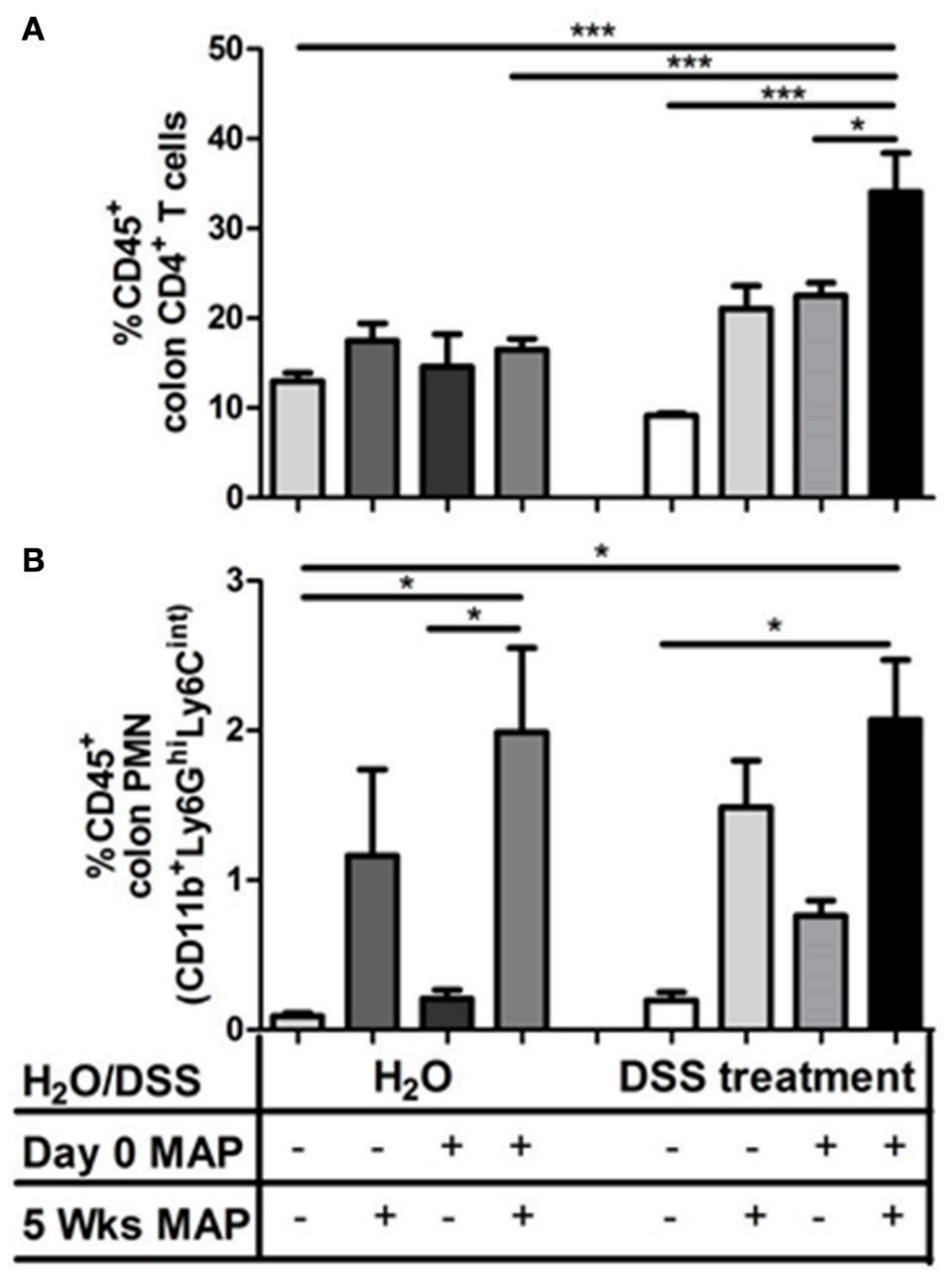
**CD4^+^ T cells accumulate in colon of DSS-treated MAP-infected mice after secondary exposure. (A)** Frequency of CD4^+^ (CD3^+^CD4^+^) and **(B)** PMN (CD11b+Ly6GhiLy6Cint) in colonic lamina propria (*n* = 3–5). Mean ± SEM. ^*^P < 0.05; ^***^p < 0.001, n.s, not significant, one-way ANOVA with Tukey's multiple comparison post-test.

To further extend these results, sera were collected additionally at 2, 6, and 24 h after secondary infection and analyzed by ELISA. Again, strongly elevated levels of TNF-α were observed at 2 h in both DSS+MAP+MAP and H_2_O+MAP+MAP mice (Figure 3A). Levels gradually decreased afterwards. Also, IFN-γ levels were elevated at 2 h, reached the peak at 6 h and decreased at 24 h after secondary challenge. Of note, IFN-γ levels remained high in mice that had received DSS prior to MAP administration (Figure S2A). Similarly elevated levels of TNF-α and IFN-γ were observed in supernatants of colonic tissue (Figure S2B). Here TNF-α levels remained elevated at 24 h in DSS+MAP+MAP mice consistent with the inflammation and diarrhea observed under these conditions.

### DSS+MAP+MAP treated mice exhibit increased MLN leukocyte numbers

Mesenteric lymph nodes (mLN) drain the intestinal mucosa and are located within the mesenteric tissue. In order to investigate the influence of DSS/MAP-induced mucosal inflammation on the local lymph node environment, flow cytometric analysis of suspended lymph node cells was applied. Only mice pretreated with DSS and receiving MAP showed a consistent increase of hematopoietic cells (Figure S3). No particular cell type was preferentially increased indicating an enhanced cell influx or reduced cell efflux.

### Role of CD4^+^ T cells in the DSS/MAP induced colon inflammation

Consistent with a contribution of CD4^+^ T cells in the induction of colitis, secondary infection led to a significant increase of CD4^+^ T cells in the colonic *lamina propria* following DSS administration in combination with repeated MAP exposure (Figure 3A). In contrast, the level of CD8^+^ T cells as well as CD19^+^ B cells remained unaltered (Figure S4). Among the myeloid cells, only PMNs showed a significant increase (Figure 3B). However, this increase was only associated with MAP administration and independent of DSS treatment and therefore not associated with the emergence of enhanced mucosal inflammation.

**Figure 4:**
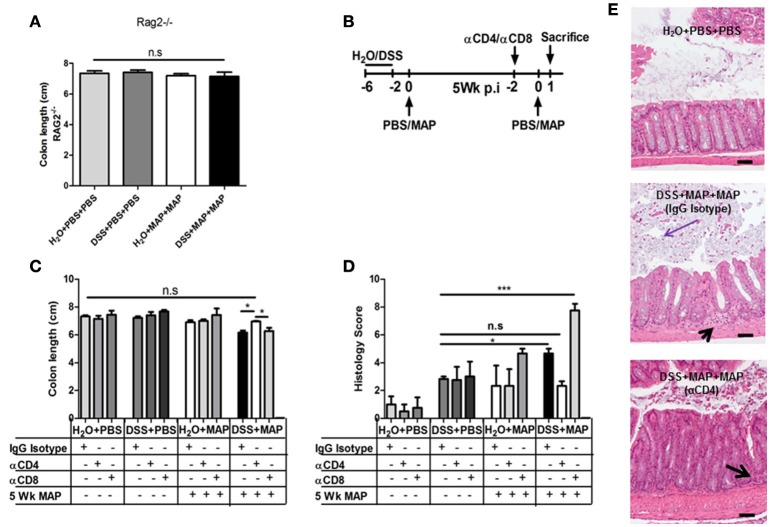
**Role of CD4^+^ T cells in colitis. (A)** Upon the necropsy at 1 day after secondary exposure, colon length of RAG^−/−^ mice was measured. Colon lengths in cm (*n* = 4–6). **(B)** Experimental scheme for CD4 and CD8 T cell depletion. **(C)** Colon Length of CD4 or CD8 depleted and IgG Isotype control mice (*n* = 3–4). **(D)** Histology score of the colon analyzed at 1 day after secondary exposure (*n* = 3–5). **(E)** H&E staining of colon tissue sections at day 1 after secondary exposure. Scale bars, 25 μm. H_2_O+PBS+PBS (upper part) showed normal tissue. DSS+MAP+MAP IgG isotype (middle) showed mild invasion of inflammatory cells (black arrow) and moderate epithelial hyperplasia with desquamation of epithelial cells into the lumen (purple arrow). DSS+MAP+MAP depleted CD4^+^ T cells (bottom) showed mild invasion of inflammatory cells (black arrow). Graph show a representative of at least two independent experiments, show as Mean ± SEM. n.s, not significant, ^*^P < 0.05; ^***^p < 0.001, one-way ANOVA with Tukey's multiple comparison post-test.

To confirm the role of CD4^+^ T cells in the MAP-mediated exacerbation of the DSS-induced colitis, we next treated RAG2^−/−^ mice, which lack T and B cells with DSS and subsequently challenged them with MAP. As expected, no reduction of colon length was observed in the absence of a functional T cell-mediated immunity (Figure 4A).

To assess the key players for this phenomenon, CD4^+^ T cells or CD8^+^ T cells from C57BL/6 WT mice were individually depleted using anti-CD4 or anti-CD8 antibodies 2 days before secondary MAP challenge (Figure 4B). Colon length reduction was not observed in mice depleted of CD4^+^ T cells (Figure 4C). Conversely, mice depleted of CD8^+^ T cells still showed a reduction of colon length (Figure 4C). Again, no colon length reduction was observed in DSS+MAP+PBS, DSS+PBS+MAP, H_2_O+MAP+PBS and H_2_O+PBS+MAP control animals (data not shown).

Consistent with these results, the histology score of colonic tissue obtained from mice depleted of CD4^+^ T cells showed a significant improvement in comparison to mice depleted of CD8^+^ T cells or isotype controls (Figure 4D). Moderate infiltration of inflammatory cells and mild epithelial hyperplasia was observed in isotype control or CD8^+^ T cell-depleted DSS+MAP+MAP mice. In contrast, colonic tissues from DSS+MAP+MAP mice depleted of CD4^+^ T cells showed only mild tissue infiltration by inflammatory cells (Figure 4E, Figure S5).

### Different immune reaction to infection with closely related mycobacterial species

**Figure 5:**
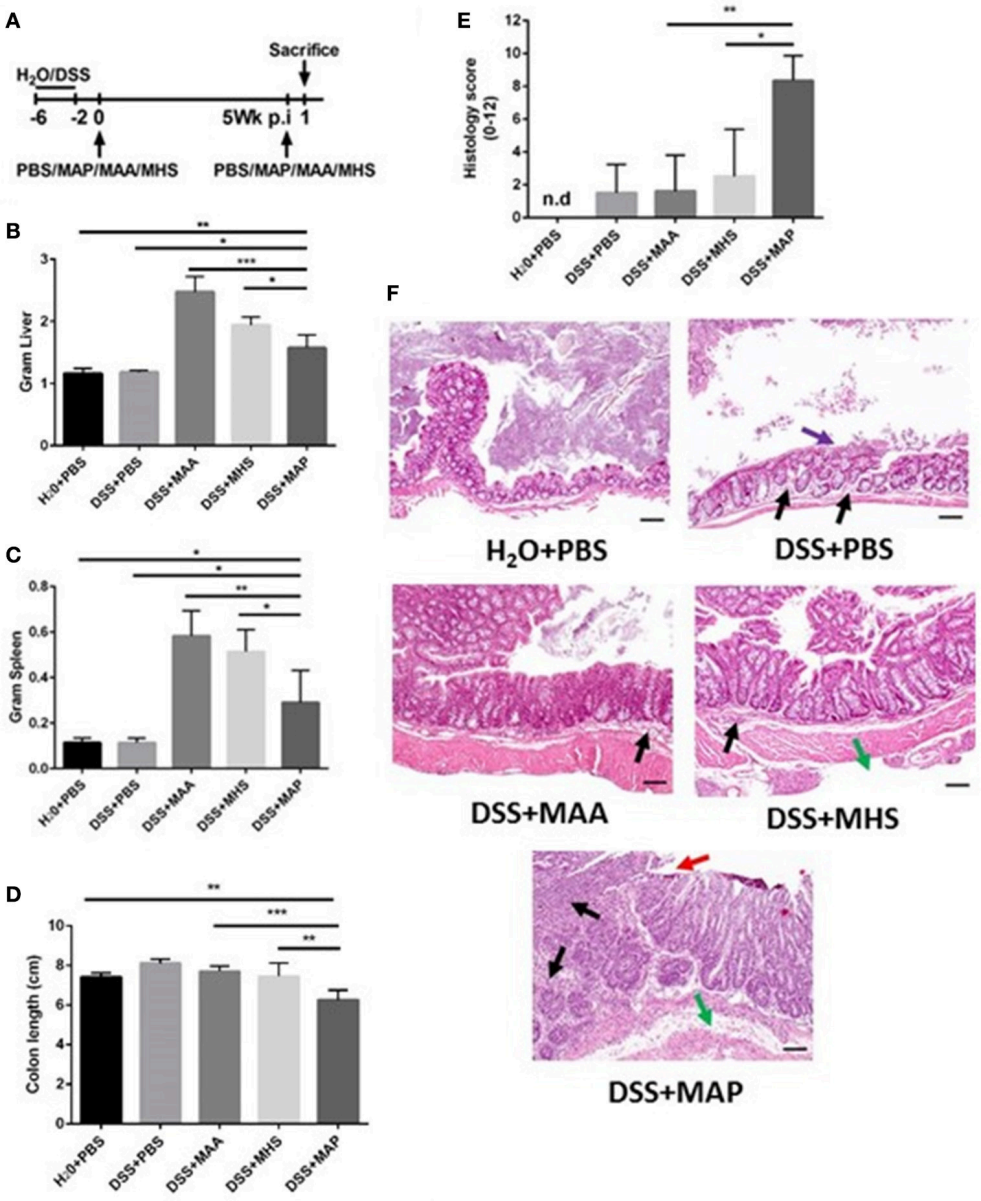
**Different responses elicited by infection closely related mycobacterial species after secondary exposure. (A)** Experimental scheme. **(B–D)** Upon necropsy at 1 day after secondary exposure, colon length, liver and spleen weight were measured. **(B)** Total liver weight in Gram. **(C)** Total spleen weight in Gram. **(D)** Colon length of MAP, MAA and MHS infected group at 1 day after secondary exposure with the same mycobacteria species (*n* = 3–5). **(E)** Histology score of the colon analyzed at 1 day after secondary exposure (*n* = 3–5). n.d = not detected, histology score = 0. **(F)** H&E staining of colon tissue sections at day 1 after secondary exposure. Scale bars, 25 μm. H_2_O+PBS+PBS (upper left) showed normal tissue. DSS+PBS+PBS (upper right) showed mild invasion of inflammatory cells (black arrow), mild epithelial hyperplasia and desquamation of epithelial cells (purple arrow). DSS+MAA+MAA (middle left) showed mild invasion of inflammatory cells (black arrow), moderate epithelial hyperplasia. DSS+MHS+MHS (middle right) showed mild invasion of inflammatory cells (black arrow), mild edema (green arrow), moderate epithelial hyperplasia. DSS+MAP+MAP (bottom part) showed severe transmural invasion of inflammatory cells (black arrow), ulceration (red arrow), moderate edema (green arrow) and severe epithelial hyperplasia. Graph show a representative of at least two independent experiments, show as Mean ± SEM. ^*^P < 0.05; ^**^p < 0.01; ^***^p < 0.001, one-way ANOVA with Tukey's multiple comparison post-test.

To investigate the immune reaction of closely related mycobacterial species, we infected DSS-treated mice with the closely related mycobacterial species *M. avium* ssp. *avium* (MAA) or *M. avium* ssp. *hominissuis* (MHS). At 5 weeks after the first challenge, a secondary challenge was administered with the same mycobacterial species (Figure 5A). All infected mice showed an increase in liver weight in comparison to uninfected animals (Figure 5B). In fact, mice infected with MAA and MHS exhibited an even higher total spleen weight in comparison to MAP infected animals (Figure 5C). In contrast, only DSS-treated and MAP infected mice showed a significant reduction of colon length after secondary MAP challenge. Neither DSS-treated MAA infected mice nor DSS-treated MHS infected animals showed this phenomenon (Figure 5D). Consistent with these results, the histology score of colon tissue obtained from DSS-treated and MAP infected mice after secondary MAP challenge showed a significant higher histology score in comparison to DSS-treated MAA or MHS infected animals after secondary challenge with the same bacteria (Figure 5E). Severe transmural invasion of inflammatory cells, ulceration, moderate edema and severe epithelial hyperplasia was observed in DSS+MAP+MAP mice. In contrast, colonic tissues from DSS+MAA+MAA and DSS+MHS+MHS showed only mild invasion of inflammatory cells and moderate epithelial hyperplasia (Figure 5F). This illustrates that high colonic inflammation was specific for MAP in comparison to the closely related mycobacteria MAA and MHS. Interestingly, despite that MAP infection showed higher inflammation, colonization by live MAA in intestine exhibited higher bacterial loads in comparison to the DSS+MAP+MAP and DSS+MHS+MHS group (Figure S6). In agreement, ZN staining of intestine showed very rarely positive signals for animals of the DSS+MAP+MAP and DSS+MHS+MHS group. High positive signals were obtained for the DSS+MAA+MAA group (Figure S7).

## Discussion

Here we provide clear evidence that recurrent exposure to MAP aggravates intestinal inflammation and clinical symptoms suggesting a disease promoting capability of MAP which might be of importance in JD and CD.

We provide conclusive evidence for the unique capacity of MAP to exacerbate DSS-mediated mucosal inflammation in the colon. We also identify the critical role of CD4^+^ T cells in this process consistent with their function in many other animal models of intestinal inflammation and human IBD (Powrie et al., 1994; Shale et al., 2013). DSS represents a well-established widely used murine IBD model. IBD-like clinical symptoms like diarrhea, bloody feces, reduction of colon length, mucosal ulceration and loss of body weight develop as a consequence of DSS administration in the drinking water (Elson et al., 1995; Wirtz et al., 2007). DSS is known to induce mucosal injury and inflammation via a direct toxic effect on enterocytes and the protective mucus layer (Johansson et al., 2010). This provides a port of entry for intestinal bacteria resulting in an inflammatory response (Saleh and Elson, 2011; Johansson et al., 2013). In this model, components of the innate immune system such as TLRs and the inflammasome are important for tissue repair and protection from colitis (Saleh and Elson, 2011). In contrast, the adaptive immune system represented by T_H_1 and T_H_2 CD4^+^ cells does not play a critical role and has been shown to only influence the inflammation during the late course of the disease (Dieleman et al., 1998). Hence, DSS was selected to cause the primary lesion. Thus, we took advantage of the DSS model to examine repeated administration of MAP. Using this model we could show that i.p. infection with MAP exacerbate an existing mucosal inflammation by its capacity to stimulate a CD4^+^ T cell response.

The observed enhanced loss in body weight and delayed recovery in mice exposed to both, DSS and MAP were moderate following a single MAP administration. These finding were consistent with our previous data (Suwandi et al., 2014). Remarkably, symptoms were significantly more pronounced after repeated challenge suggesting aggravation of inflammation. Therefore, the pronounced reduction of body weight after repeated challenge with MAP was most likely due to the high cytokine and/or chemokine levels secreted by adaptive immune cells in response to secondary mycobacterial exposure. However, the bacteria-induced immune stimulation could not be the sole explanation, since the clinical course in DSS+MAP+MAP mice was much more severe than in H_2_O+MAP+MAP treated animals. Rather, the DSS-induced preexisting intestinal inflammation significantly contributed to the observed phenotype. Thus, the immune response elicited either by MAP itself or by MAP-induced host components, exacerbated the DSS-mediated tissue damage. Strikingly, this exacerbation even led to diarrhea, a symptom rarely observed in mice. In addition, it led to a strong local inflammatory reaction illustrated by a reduction of colon length and the infiltration of immune cells resembling the clinical picture of CD or JD. Interestingly, in our current study as well as in our previous work (Suwandi et al., 2014), low colonization of the intestine by MAP was observed. In accordance, Ziehl-Neelsen (ZN) staining did only very rarely reveal positive signals.

Functionally, a significant reduction of fluid absorption at the colon mucosa was observed after secondary challenge with MAP even in the absence of DSS-pretreatment. Similarly, also the MAP induced increase in TNF-α and IFN-γ in serum or in colon tissue supernatants was independent of preexisting DSS-mediated inflammation. In accordance, a previous study reported that administration of antagonistic anti-CD3 antibodies was able to prevent net water absorption. Such anti-CD3 treatment also caused systemic cytokine release and acute TNF-dependent diarrhea (Musch et al., 2002; Clayburgh et al., 2005). Hence, we assume that both the innate immune system as well as memory cells of the adaptive immune system react to the repeated MAP exposure. Particularly CD4^+^ T cells might be stimulated to secrete cytokines such as TNF-α and IFN-γ leading to a reduction of fluid absorption and diarrhea.

Most likely, different cellular mechanisms are responsible for the inflammatory reaction and the shortening of the colon. Analysis of the draining MLN did not reveal a clear increase of a particular cell type irrespective of the type of stimulation. In contrast, an increased frequency of PMNs was observed in the *lamina propria* of the colon. This increase, however, was observed in all groups receiving MAP administration. Thus, the recruitment of PMNs is most likely owed solely to the MAP exposure. In contrast, the increased frequency of inflammatory monocytes in all DSS-treated and MAP infected mice was probably due to the primary lesion. Since recruitment of inflammatory monocytes is essential for controlling and clearing bacterial infections (Shi and Pamer, 2011), the presence of such cells might suggest an ongoing translocation of commensal bacteria derived from the enteric microbiota.

The reduction of colon length in the DSS-treated and repeatedly MAP infected group required CD4^+^ T cells as shown by depletion experiments. In accordance, this cell population was up-regulated in the colon tissue of DSS+MAP+MAP mice. CD4^+^ T cells are known to play an important role during mycobacterial infections (Ehlers and Schaible, 2012). The presence of CD4^+^ T cells accelerates the granulomatous anti-mycobacterial response by an enhanced production of TNF-α and IFN-γ at the site of infection (Hänsch et al., 1996). On the other hand, CD4^+^ T cells are also important in the pathogenesis of colitis. Clonal T cell populations specific for bacterial antigens can induce colitis (Kullberg et al., 2003). In addition, CD4^+^ T cells with high expression of CD45RB are able to induce pathogenic T_H_1 responses that lead to colitis (Powrie et al., 1994). Based on our results, we suggest that MAP specific CD4^+^ T cells are activated upon repeated challenge. This induces the maturation of colitogenic effector T cells. The stronger inflammatory effect in DSS-treated mice illustrated by the reduction in colon length and elevated histological score might result from a synergistic effect between mycobacteria-specific T cell responses, the acute innate immune response to mycobacterial constituents and the underlying tissue impairment caused by DSS treatment.

Interestingly, mice exposed to both, DSS and MAP exhibited symptoms of severe intestinal inflammation. In contrast, the closely related mycobacteria MAA and MHS caused milder intestinal inflammation in combination with DSS, despite of their potent immune-activating activity illustrated by the enhanced liver and spleen tissue weight. In addition, MAA colonized the intestine (small intestine and colon) in high numbers as revealed by plating and ZN staining. Severely reduced numbers of MAP and MHS were observed under these conditions by plating while ZN staining rarely revealed positive signals. Most likely high proliferation of MAA is responsible for the high colonization although MHS might proliferate but could be controlled by the immune system. Thus, far, we never found evidence for proliferation of MAP in our animal models.

The differential induction of intestinal inflammation by the three mycobacteria might be due to a particular cell tropism of MAP to myeloid cells of the intestine i.e., a particular localization in the colon which could not be revealed by histology, Alternatively, MAP might induce a CD4^+^ T cell response to self-components or constituents of the enteric microbiota driving colitis in individuals suffering from an underlying mucosal disease. This clearly needs more intensive studies in the future. However, the overall results would be in accordance with our original hypothesis that MAP exacerbates a pre-existing injury of the intestine and suggests that MAP might represent an aggravating factor in the development of IBD. In conclusion, our results demonstrate a strong mucosal immune response to repeated MAP infection in animals with a preexisting colon disease. The repeated challenge with MAP causes re-activation of CD4^+^ T cells leading to a severe mucosal inflammation. In addition, it induces a massive cytokine release that is known to lead to diarrhea. Thus, at least two mechanisms appear to contribute to disease severity: first, impairment of the epithelial barrier function likely due to cytokine secretion, and second, a MAP stimulated immune cell activation that amplifies the cytokine production and, together with the preexisting tissue impairment, aggravates the mucosal inflammation resembling IBD. Importantly, both phenomena result in typical clinical symptoms seen in JD and CD. Thus, the presented model provides important molecular and cellular clues on the association of MAP infection in intestinal inflammation.

## Author contributions

AS, IB, TB, CF, US, MH, RG, SW designed experiments. AS, MP, MK, SZ, AKS performed experiments. AS, MH, RG, SW analyzed the data. AS, MH, RG, SW wrote the manuscript.

## Funding

This work was partly funded by the ZooMAP program of the German Federal Ministry of Education and Research (BMBF, ZOOMAPII: 01KI1003B and 01KI1003C), SFB621/C9, DFG-SE460/13-4 and the German Center for Infection Research (DZIF), partner site Hannover-Braunschweig, TTU- IICH (01/2013-12/2015).

### Conflict of interest statement

The authors declare that the research was conducted in the absence of any commercial or financial relationships that could be construed as a potential conflict of interest.

## Supplementary material

Supplementary Figure 1 **Body weight measurement. (A–D)** Weight change during experiment, expressed as percentage change from day 1 of DSS treatment. **(A)** Graph shown from whole experiment. **(B)** The graph shown is only from 5 week after first exposure. **(C)** Graph shown % body weigh measurement at day 0, 1, and 2 post first exposure. **(D)** Graph shown % body weight measurement at day 0, 1, 2, and 4 post secondary exposure at 5 weeks. Graph show a representative of more than three independent experiments (*n* = 4–5). ^*^*P* < 0.05; ^***^*p* < 0.001; n.s, not significant, Mann-Whitney test.

Supplementary Figure 2 **Elevated cytokine levels after secondary administration of MAP. (A,B)** Kinetics of TNF-α and IFN-γ in serum and colon supernatant at 2, 6, and 24 h after secondary challenge (*n* = 3–5). **(A)** Cytokines level in serum **(B)** secretion of cytokines into the supernatant of overnight cultured colon, show as Mean ± SEM. n.d = not detected.

Supplementary Figure 3 **Cellularity in mesenteric lymph nodes. (A,B)** Flow cytometry on leukocytes in mLN at 1 day after secondary exposure. **(A)** Flow cytometry gating strategy for myeloid cells and lymphocytes from mLN. **(B)** Total cell numbers of CD4+ (CD3+CD4+) and CD8+ (CD3+CD8+) T cells, CD19+ B cells, dendritic cells (CD11b+CD11chi), PMN (CD11b+Ly6GhiLy6Cint), monocytes (CD11b+Ly6ChiLy6Gint) and macrophage (CD11b+ Ly6Glo Ly6CloF4/80lo) (*n* = 3–5). Graph show a representative of at least two independent experiments. ^*^*P* < 0.05; ^**^*p* < 0.01; ^***^*p* < 0.001, one-way ANOVA with Tukey's multiple comparison post-test.

Supplementary Figure 4 **Cellularity in colonic lamina propria. (A,B)** Flow cytometry on leukocytes in colonic lamina propria at 1 day after secondary exposure. **(A)** Flow cytometry gating strategy for myeloid cells and lymphocytes from colonic lamina propria. **(B)** Frequency of CD19+ B cells, CD4+ (CD3+CD4+), and CD8+ (CD3+CD8+) T cells, dendritic cells (CD11b+ CD11chi), PMN (CD11b+Ly6GhiLy6Cint), inflammatory monocytes (CD11b+Ly6ChiLy6Gint) and macrophages (CD11b+Ly6GloLy6CloF4/80hi) in colonic lamina propria (*n* = 3–5). ^*^*P* < 0.05; ^**^*p* < 0.01; ^***^*p* < 0.001, n.s, not significant, one-way ANOVA with Tukey's multiple comparison post-test.

Supplementary Figure 5 **Role of CD4+ T cells in colitis**. H&E staining of colon tissue sections at day 1 after secondary exposure. Scale bars, 25 μm. H2O+PBS+PBS (upper left) showed normal colonic tissue. DSS+PBS+PBS, DSS+PBS+MAP, DSS+MAP+PBS, H2O+MAP+MAP, and DSS+MAP+MAP depleted CD4+ T cells (bottom second from the left) showed mild invasion of inflammatory cells (black arrow). DSS+MAP+MAP IgG isotype (upper right) showed mild invasion of inflammatory cells (black arrow), moderate epithelial hyperplasia with desquamation of epithelial cells into the lumen (purple arrow showing epithelial desquamation). DSS+MAP+MAP depleted CD8+ T cells showed moderate invasion of inflammatory cells (blue arrow), moderate erosion of goblet cells and epithelial cells (green arrow), and desquamation of epithelial cells into the gut lumen (purple arrow showing epithelial desquamation).

Supplementary Figure 6 **Viable bacteria in intestine by infection closely related mycobacterial species after secondary exposure**. Bacterial loads in intestine (CFU/g intestine) were analyzed at 1 day after secondary exposure with the same mycobacteria species by plating intestine on Middlebrook Agar (*n* = 3–5). ^***^*p* < 0.001, one-way ANOVA with Tukey's multiple comparison post-test.

Supplementary Figure 7 **ZN staining of organs by infection closely related mycobacterial species after secondary exposure**. ZN staining of colon (upper part), mesentery (middle part) and small intestine (bottom part) of DSS+MAP+MAP (left), DSS+MAA+MAA (middle), and DSS+MHS+MHS (right) groups, showed high positive signal (black arrow) in mesentery of all groups. Only in DSS+MAA+MAA group revealed high positive signal (black arrow) in colon (upper part in the middle) and small intestine (bottom part in the middle), which did not reveal in DSS+MAP+MAP and DSS+MHS+MHS. Scale bars, 25 μm.

Supplementary Table 1 **Cytokines and chemokines level using multiplex assay at 0 and 6 h post second infection**. Data are presented in pg/mL. OOR, Out of Range; OOR>, Out of Range Above; OOR <, Out of Range Below.
