# Supplementary figures and images for: CD4 T Cell Dependent Colitis Exacerbation Following Re-Exposure of Mycobacterium avium ssp. paratuberculosis
Source: Front Cell Infect Microbiol. 2017 Mar 16;7:75. doi: 10.3389/fcimb.2017.00075 (PMC5352692; doi:10.3389/fcimb.2017.00075)

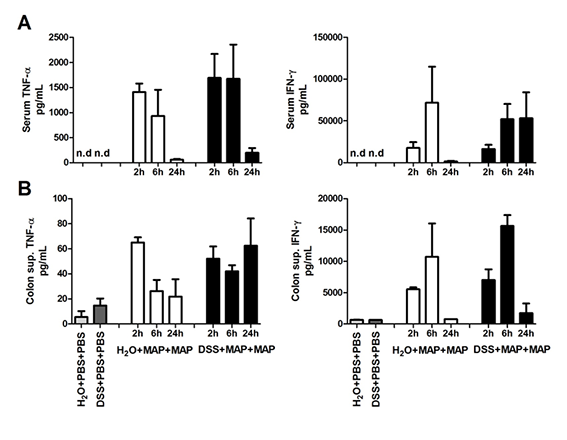

Supplement: Supplementary Figure 2 — Elevated cytokine levels after secondary administration of MAP. (A,B) Kinetics of TNF-α and IFN-γ in serum and colon supernatant at 2, 6, and 24 h after secondary challenge (n = 3–5). (A) Cytokines level in serum (B) secretion of cytokines into the supernatant of overnight cultured colon, show as Mean ± SEM. n.d = not detected. [file Image2.TIF]

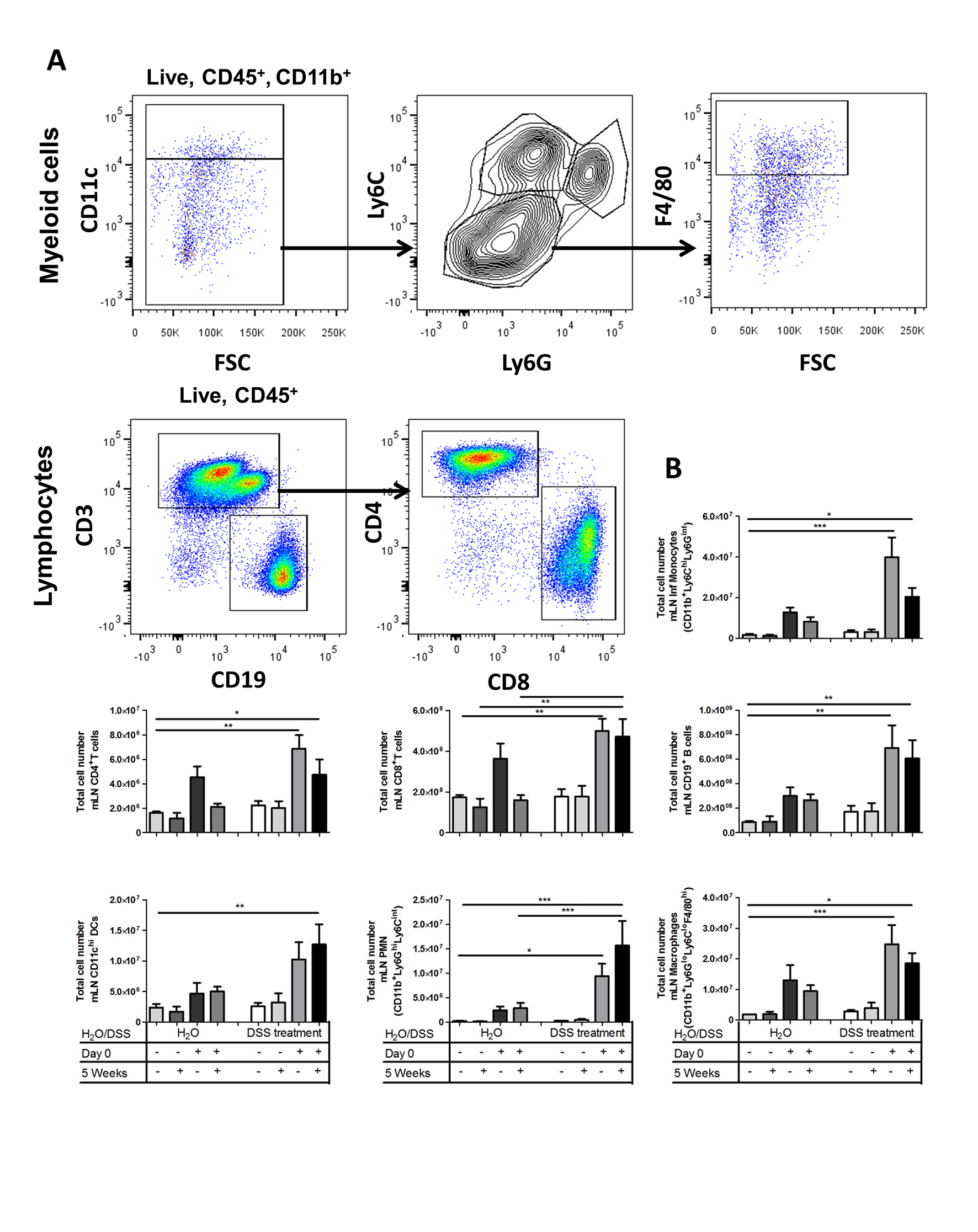

Supplement: Supplementary Figure 3 — Cellularity in mesenteric lymph nodes. (A,B) Flow cytometry on leukocytes in mLN at 1 day after secondary exposure. (A) Flow cytometry gating strategy for myeloid cells and lymphocytes from mLN. (B) Total cell numbers of CD4+ (CD3+CD4+) and CD8+ (CD3+CD8+) T cells, CD19+ B cells, dendritic cells (CD11b+CD11chi), PMN (CD11b+Ly6GhiLy6Cint), monocytes (CD11b+Ly6ChiLy6Gint) and macrophage (CD11b+ Ly6Glo Ly6CloF4/80lo) (n = 3–5). Graph show a representative of at least two independent experiments. *P < 0.05; **p < 0.01; ***p < 0.001, one-way ANOVA with Tukey's multiple comparison post-test. [file Image3.TIF]

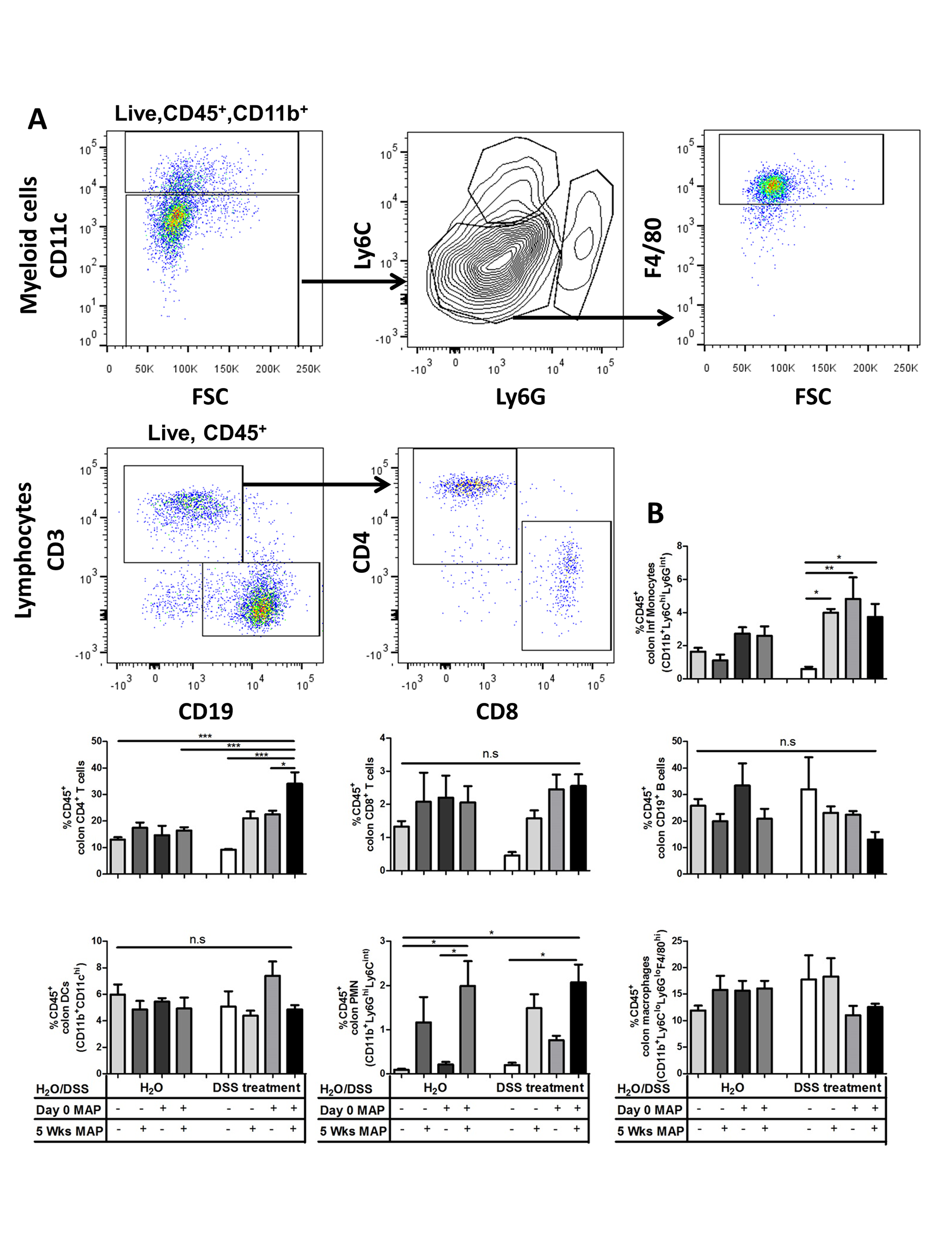

Supplement: Supplementary Figure 4 — Cellularity in colonic lamina propria. (A,B) Flow cytometry on leukocytes in colonic lamina propria at 1 day after secondary exposure. (A) Flow cytometry gating strategy for myeloid cells and lymphocytes from colonic lamina propria. (B) Frequency of CD19+ B cells, CD4+ (CD3+CD4+), and CD8+ (CD3+CD8+) T cells, dendritic cells (CD11b+ CD11chi), PMN (CD11b+Ly6GhiLy6Cint), inflammatory monocytes (CD11b+Ly6ChiLy6Gint) and macrophages (CD11b+Ly6GloLy6CloF4/80hi) in colonic lamina propria (n = 3–5). *P < 0.05; **p < 0.01; ***p < 0.001, n.s, not significant, one-way ANOVA with Tukey's multiple comparison post-test. [file Image4.TIF]

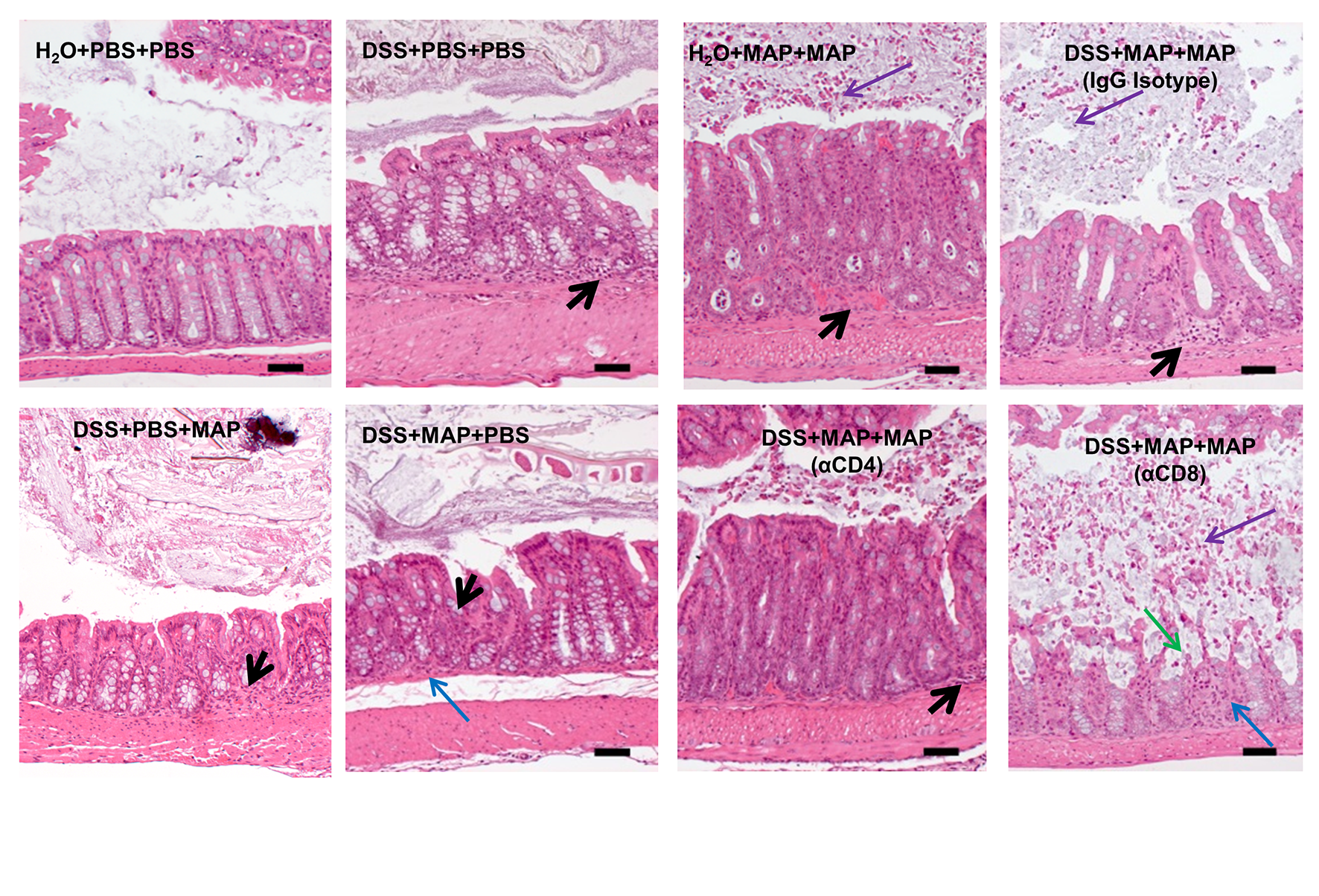

Supplement: Supplementary Figure 5 — Role of CD4+ T cells in colitis. H&E staining of colon tissue sections at day 1 after secondary exposure. Scale bars, 25 μm. H2O+PBS+PBS (upper left) showed normal colonic tissue. DSS+PBS+PBS, DSS+PBS+MAP, DSS+MAP+PBS, H2O+MAP+MAP, and DSS+MAP+MAP depleted CD4+ T cells (bottom second from the left) showed mild invasion of inflammatory cells (black arrow). DSS+MAP+MAP IgG isotype (upper right) showed mild invasion of inflammatory cells (black arrow), moderate epithelial hyperplasia with desquamation of epithelial cells into the lumen (purple arrow showing epithelial desquamation). DSS+MAP+MAP depleted CD8+ T cells showed moderate invasion of inflammatory cells (blue arrow), moderate erosion of goblet cells and epithelial cells (green arrow), and desquamation of epithelial cells into the gut lumen (purple arrow showing epithelial desquamation). [file Image5.TIF]

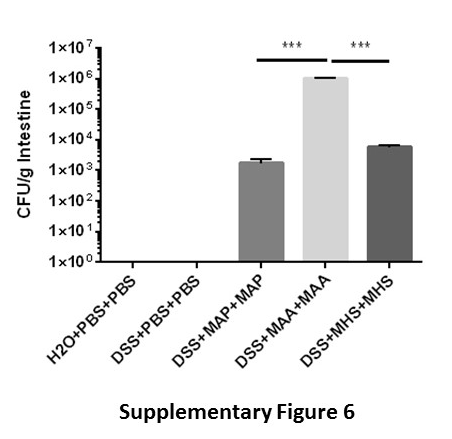

Supplement: Supplementary Figure 6 — Viable bacteria in intestine by infection closely related mycobacterial species after secondary exposure. Bacterial loads in intestine (CFU/g intestine) were analyzed at 1 day after secondary exposure with the same mycobacteria species by plating intestine on Middlebrook Agar (n = 3–5). ***p < 0.001, one-way ANOVA with Tukey's multiple comparison post-test. [file Image6.TIF]

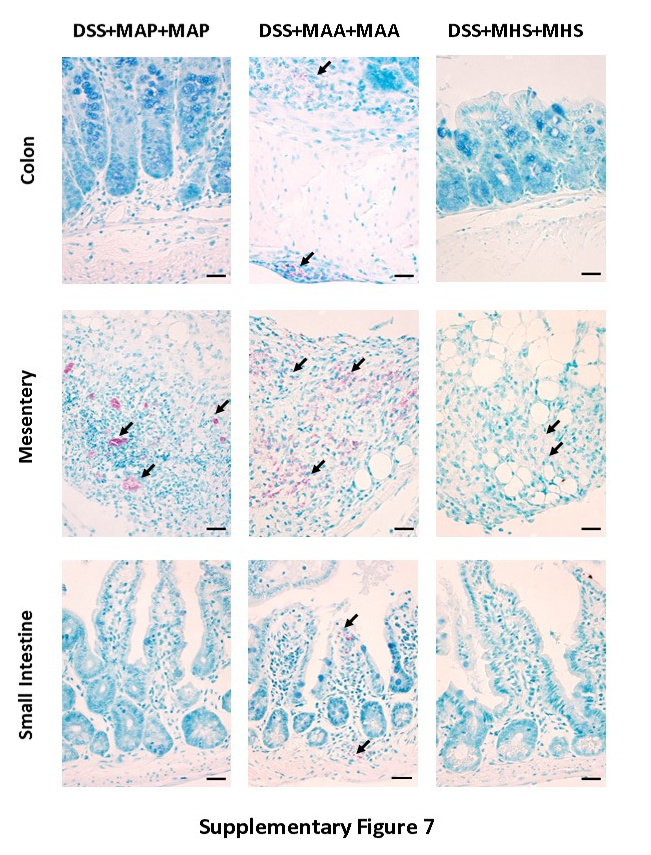

Supplement: Supplementary Figure 7 — ZN staining of organs by infection closely related mycobacterial species after secondary exposure. ZN staining of colon (upper part), mesentery (middle part) and small intestine (bottom part) of DSS+MAP+MAP (left), DSS+MAA+MAA (middle), and DSS+MHS+MHS (right) groups, showed high positive signal (black arrow) in mesentery of all groups. Only in DSS+MAA+MAA group revealed high positive signal (black arrow) in colon (upper part in the middle) and small intestine (bottom part in the middle), which did not reveal in DSS+MAP+MAP and DSS+MHS+MHS. Scale bars, 25 μm. [file Image7.TIF]
