# Supplementary material for: CD4 T Cell Dependent Colitis Exacerbation Following Re-Exposure of Mycobacterium avium ssp. paratuberculosis
Source: Front Cell Infect Microbiol. 2017 Mar 16;7:75. doi: 10.3389/fcimb.2017.00075 (PMC5352692; doi:10.3389/fcimb.2017.00075)
Supplement: Supplementary Table 1 — Cytokines and chemokines level using multiplex assay at 0 and 6 h post second infection. Data are presented in pg/mL. OOR, Out of Range; OOR>, Out of Range Above; OOR <, Out of Range Below. [file Table1.docx]

**Supplementary Information**

**Supplementary Table 1:** Cytokines and chemokines level using multiplex assay at 0h and 6h post second infection. Data are presented in pg/mL.

Note: OOR = Out of Range; OOR> = Out of Range Above; OOR< = Out of Range Below

|  | H_2_O+PBS+PBS 6h | DSS+ PBS+PBS 6h | H_2_O+MAP 0h | DSS+MAP 0h | H_2_O+MAP+MAP 6h | DSS+MAP+MAP 6h |
| --- | --- | --- | --- | --- | --- | --- |
| IL-1α | 14,7 | 6,1 | 1,16 | 10,49 | 72,11 | 73,58 |
| IL-1β | 27,28 | 27,28 | 90,99 | 45,50 | 667,15 | 457,52 |
| IL-2 | 4,67 | 4,67 | 6,49 | 11,43 | 8284,91 | 9110,05 |
| IL-3 | OOR < | OOR < | OOR < | OOR < | 3,16 | 3,02 |
| IL-4 | 0,08 | 0,08 | 0,91 | OOR < | 3,93 | 4,10 |
| IL-5 | 38,84 | 18,59 | 12,71 | 9,46 | 496,5 | 259,48 |
| IL-6 | 2,48 | 2,34 | 2,91 | 3,48 | 8948,15 | OOR > |
| IL-9 | 22,62 | OOR < | OOR < | 17,77 | 129,2 | 169,9 |
| IL-10 | 17,49 | 8,53 | 6 | 12,36 | 350,41 | 449,54 |
| IL-12(p40) | 77,24 | 55,17 | 189,15 | 219,78 | 841,72 | 1169,19 |
| IL-12 (p70) | 20,47 | 13,34 | 14,32 | 17,24 | 69,38 | 83,26 |
| IL-13 | 15,43 | 7,44 | 27,53 | 60,21 | 235,72 | 218,82 |
| IL-17 | 34,66 | 24,8 | 19,22 | 26,59 | 336,4 | 180,62 |
| Eotaxin/ CCL11 | 112,92 | 45,98 | OOR < | 36,76 | 2245,66 | 5007,7 |
| G-CSF | 148,21 | 208,14 | 49,56 | 970,22 | 16694,29 | 28297,56 |
| GM-CSF | 11,87 | OOR < | OOR < | OOR < | 112,3 | 129,9 |
| IFN-g | 2,32 | 0,45 | 2,74 | 2,74 | 1505,6 | 2467,31 |
| KC/ CXCL1 | 24,72 | 10,02 | 20,73 | 50,78 | 8048,34 | 7671,89 |
| MCP-1/ CCL2 | 75,69 | 65,42 | 166,46 | 117,67 | 10854 | 11987,42 |
| MIP-1a/ CCL3 | 2,22 | 0,94 | 4,70 | 9,09 | 215,64 | 227,95 |
| MIP-1b/ CCL4 | 12,83 | 8,52 | 9,27 | 13,51 | 274,63 | 518,22 |
| RANTES/ CCL5 | 14,73 | 11,8 | 41,95 | 39,53 | 391,88 | 502,89 |
| TNF-α | 793,64 | 339,9 | 681,24 | 961,33 | 5005,62 | 4209,94 |
